# Transcriptional responses of PBMC in psychosocially stressed animals indicate an alerting of the immune system in female but not in castrated male pigs

**DOI:** 10.1186/1471-2164-15-967

**Published:** 2014-11-08

**Authors:** Michael Oster, Eduard Muráni, Siriluck Ponsuksili, Richard B D’Eath, Simon P Turner, Gary Evans, Ludger Thölking, Esra Kurt, Ronald Klont, Aline Foury, Pierre Mormède, Klaus Wimmers

**Affiliations:** Leibniz Institute for Farm Animal Biology (FBN), Institute for Genome Biology, Wilhelm-Stahl-Allee 2, 18196 Dummerstorf, Germany; Animal & Veterinary Science Research Group, SRUC, West Mains Road, Edinburgh, EH9 3JG UK; PIC UK, 2 Kingston Business Park, Kingston Bagpuize, Oxfordshire, OX13 5FE UK; PIC Germany, PIC Deutschland GmbH, Ratsteich 31, 24837 Schleswig, Germany; Optimeter, Oyaksitesi 1.kisim 11b blok da:4, Sefakoy, Istanbul, Turkey; Vion Food Group, Boseind 10, 5281 RM Boxtel Postbus 1, 5280 AA Boxtel, The Netherlands; Université Victor Segalen Bordeaux 2, PsyNuGen, UMR 1286 INRA, 33076 Bordeaux, France

**Keywords:** Immune system, Microarray, Peripheral blood mononuclear cells (PBMC), Pigs, Psychosocial stress, Unfamiliar mixing

## Abstract

**Background:**

Brain and immune system are linked in a bi-directional manner. To date, it remained largely unknown why immune components become suppressed, enhanced, or remain unaffected in relation to psychosocial stress. Therefore, we mixed unfamiliar pigs with different levels of aggressiveness. We separated castrated male and female pigs into psychosocially high- and low- stressed animals by skin lesions, plasma cortisol level, and creatine kinase activity obtained from agonistic behaviour associated with regrouping. Peripheral blood mononuclear cells (PBMC) were collected post-mortem and differential gene expression was assessed using the Affymetrix platform (n = 16).

**Results:**

Relevant stress-dependent alterations were found only between female samples, but not between castrated male samples. Molecular routes related to TREM 1 signalling, dendritic cell maturation, IL-6 signalling, Toll-like receptor signalling, and IL-8 signalling were increased in high stressed females compared to low stressed females. This indicates a launch of immune effector molecules as a direct response. According to the shifts of transcripts encoding cell surface receptors (e.g. *CD14*, *TLR2*, *TLR4*, *TREM1*) the study highlights processes acting on pattern recognition, inflammation, and cell-cell communication.

**Conclusions:**

The transcriptional response partly affected the degree of ‘stress responsiveness’, indicating that the high stressed females altered their signal transduction due to potential infections and injuries while fighting.

**Electronic supplementary material:**

The online version of this article (doi:10.1186/1471-2164-15-967) contains supplementary material, which is available to authorized users.

## Background

The brain is seen as superior organ which perceives psychosocial stress and orchestrates subsequently physiological stress responses. In this context, as reviewed elsewhere the bi-directional link between the brain and the immune system is of particular interest [1–3]. There are clues about evolutionary highly conserved mechanisms organising the response to acute stress, including an increased expression of genes contributing to an effective infection defence, despite these alterations have previously been thought of as rather secondary effects [4]. Because many acutely stressful events do also likely result in an increased number of injuries such as skin lesions [5, 6], stress responses are thought to involve immune alterations in general [7].

In pigs, social mixing results in psychosocial stress and aggressive behaviour including fighting [5, 6, 8]. Their severity can be indirectly measured using skin lesion counts, cortisol levels, and creatine kinase activity [5, 9, 10]. This could serve as a model to study the interrelation of psychosocial stress, aggressive behaviour, and physiological responses. In pigs the relationship between psychosocial stress, neuroendocrine factors and immune status has been the focus of only a few scientific studies using a variety of different stressors. In response to stress, some aspects of immune status became suppressed, enhanced, or remained unaffected. For example, in piglets a 2 h daily social isolation caused a reduction in lymphocyte proliferation [11], whereas 14 d of crowding and heat caused an increased NK cytotoxicity [12]. Further, social status was identified to be important with respect to lymphocyte proliferation and antibody production [13, 14]. Whereas social stress due to crowding decreased cortisol levels [12], mixing stress either increased cortisol levels [5] or remained them unaltered in dominant pigs [14]. These inconsistent findings may reflect the need to distinguish between acute and chronic stressors and other factors like age, social status and genetics. Accordingly, a genetic component of post-mixing aggressiveness was estimated in grower-stage pigs [10, 15, 16]. Further, there are a few microarray experiments which provide insight into transcriptional responses following psychosocial stress in adrenal gland [17] and liver tissue [18]. It appeared that psychosocial stress provoked expression patterns similar to those induced by ACTH stimulation. In particular, psychosocially high-stressed animals altered transcripts associated with catecholamine degradation, energy mobilizing processes, cholesterol accumulation and cholesterol biosynthesis. Furthermore, the analyses identified transcripts which may have responded to sympathoadrenal stimulation (e.g. *GAL*, *GALP*).

In our current study, the skin lesion score and stress parameters following a mixing experiment [5] were considered to reflect individual psychosocial stress levels. We investigated transcriptional responses in peripheral blood mononuclear cells (PBMC), a tissue representing a critical component of the immune system. Gene expression profiles of animals divergent for psychosocial stress level indicate an alerting of the immune system in female pigs but not in castrated males.

## Methods

### Animals, balanced mixing, sample collection

**Figure 1:**
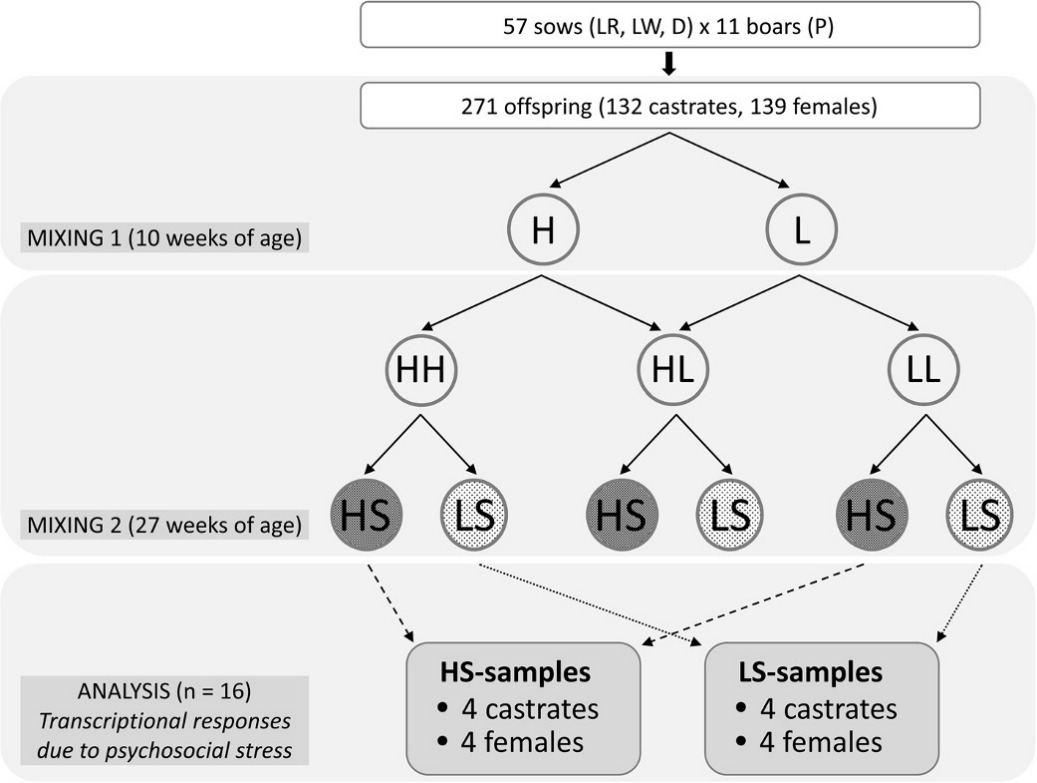
**Experimental design used to analyse transcriptional responses due to different levels of psychosocial stress.** The origin and composition of the profiled sampling groups is shown regarding their parental genetics, affiliation to skin lesion group, and resulted stress levels. LR - Landrace; LW – Large White; D – Duroc; P – Pietrain; H – High lesion score; L – Low lesion score; HS – High stress; LS – Low stress.

In order to identify pigs differing in their stress level, a mixing experiment was conducted which was described recently in detail by D’Eath *et al.* [5]. Animal care and tissue collection procedures followed the guidelines of the German Law of Animal Protection, and the experimental protocol was approved by the Institutional Animal Care and Use Committee (IACUC) of the Leibniz Institute for Farm Animal Biology (FBN). This study was based on phenotypic records and gene expression profiling done with castrated male and female finishing pigs. In fact, progeny (n = 271) derived from a crossbreed including Landrace, Large White, Duroc (sows) and Pietrain (boars) was bred (Figure 1) and reared commercially on slatted floors. Male animals were castrated at 4 days post-natum. At approximately 10 weeks of age, the pigs were assigned to new single-sex groups of eight or ten animals (Figure 1: MIXING 1). These groups were balanced for weight and unfamiliarity in order to minimize these determinants of aggressiveness in pigs [19, 20]. Thereby, standardized weight and unfamiliarity did likely contribute to uncover aggressive behaviour to a larger extent. Animals were housed in standardized conditions. Immediately before and at 24 hrs after mixing, skin lesions were counted. Thereby, the body was divided into front (head, neck, shoulder, and front legs), middle (flanks, and back) and rear (rump, hind legs, and tail) sections [9]. Changes in skin lesions pre- to post-mixing reflect the involvement in fighting and aggressive behaviour [9, 10]. For each mixing group the cut-off criteria for the total lesion score was individually calculated (pre- to post-mixing). Thereby, also the distribution of skin lesion scores in each group was estimated. Half of the pigs were designated as high skin lesion score group (H) (those with front lesions above average; mean in H: 60.6 ± 23.4) and the remaining half was designated as low skin lesion score group (L) (those with front lesions below average, mean in L: 27.5 ± 12.4), respectively. The first mixing revealed a mean total skin lesion score (mean ± SEM) of 140.9 ± 57.5 in H animals and of 98.6 ± 39.2 in L animals, respectively. The pigs remained in the established mixing groups until they reached 110 kg live weight corresponding to approximately 27 weeks of age. Here, the pigs were assigned to mixing groups based on their previous skin lesion score group (Figure 1: MIXING 2), as they were loaded onto a vehicle for a 270 km transport to the abattoir. In detail, single-sex groups were built by mixing four pigs from one rearing group and four pigs from another rearing group. Thus, H pigs were mixed with H pigs resulting in a HH batch, H pigs were mixed with L pigs resulting in a HL batch, or L pigs were mixed with L pigs resulting in a LL batch. Skin lesions were counted before mixing and after slaughter on the carcass and the skin lesion score was calculated (Table 1). In all these batches animals with high and low lesion scores were observed. Apparently, high and low levels of psychosocial stress (HS – High stress; LS – Low stress) were induced independently from the initial mixing group. In detail, the second mixing revealed a skin lesion score (mean ± SEM) on HS and LS animals originated from the HH mixing of 164.3 ± 42.7 and 34.5 ± 17.6, respectively, and a skin lesion score on HS and LS animals originating from LL mixing of 99.8 ± 44.1 and 24.0 ± 4.1, respectively. In order to limit bias due to circadian fluctuation of cortisol levels, pigs were slaughtered between 0600 and 0800 h in the next morning. Pigs were moved from lairage pens and stunned by means of CO_2_ gas and exsanguinated. Trunk blood was collected and stored on ice. Based on parameters of stress levels (Table 1), those animals were selected for gene expression profiling which represented extremes within mixing groups (Figure 1: ANALYSIS). Overall, the experimental design covered four slaughter days. Animals selected for microarray analyses were balanced for slaughter day. Each sampling group was represented by 4 castrates and 4 females.

**Table 1:** **Phenotype data of the analysed animals**

Parameter	HSF	HSM	LSF	LSM	p-value #
Skin lesions §					
Front	72.0 ± 16.5	65.0 ± 22.0	7.5 ± 1.3	16.0 ± 6.8	<0.001
Mid	60.0 ± 23.0	45.8 ± 22.5	12.8 ± 3.0	19.5 ± 11.7	<0.05
Rear	28.0 ± 16.9	7.8 ± 2.6	5.5 ± 1.3	8.5 ± 2.5	>0.10
Total	160.0 ± 47.9	118.5 ± 46.7	25.8 ± 3.8	44.0 ± 13.2	<0.01
Creatinekinase (U/l)	11543.0 ± 3270.5	9232.0 ± 1430.8	2062.5 ± 356.5	2904.5 ± 260.5	<0.001
Cortisol (ng/ml)	65.2 ± 8.50	72.5 ± 6.3	50.7 ± 6.7	59.0 ± 7.0	<0.10

Values are displayed as mean ± SEM; n =8; balanced for mixing groups; # effects for stress level but not for sexes; p-value (2 tailed) of a two sample t-test reflecting differences between animals with high stress level and low stress level; § skin lesions in body sections: front (head, neck, shoulders, and front legs), middle (flanks and back), and rear (rump, hind legs, and tail).

### Physiological parameters

Cortisol levels were measured with the automated analyser Centaur (Siemens Healthcare Diagnostics S.A.S., Saint Denis, France). A kit designed for human serum that we validated for pig serum was used. The intra- and inter-assay coefficients of variation (%) were 3.4 and 8.0, respectively. The creatine kinase activity was measured with a clinical biochemistry automat (COBAS-MIRA Plus, Roche Diagnostics). The intra- and inter-assay coefficients of variation (%) were 1.04 and 1.44, respectively. Both progesterone and 17β-Estradiol were analysed with a commercial enzyme-linked immunosorbent assay (ELISA) purchased from IBL International GmbH, Hamburg, Germany.

### RNA isolation, target preparation and hybridization

In total, 16 samples (balanced for mixing group) were selected for subsequent analyses. Using 4 ml of blood, peripheral blood mononuclear cells (PBMC) were isolated by centrifugation on a Histopaque (Sigma-Aldrich, Taufkirchen, Germany) density gradient. Total RNA was isolated from individual PBMC-samples using the column-based NucleoSpin RNA II Kit (MACHEREY-NAGEL, Düren, Germany). RNA integrity was checked by a 2100 Bioanalyser (Agilent) and agarose gels containing ethidium bromide. RNA concentration was measured by a NanoDrop ND-1000 spectrometer (PEQLAB, Erlangen, Germany). The absence of genomic DNA was checked by a PCR amplification of the porcine GAPDH gene (forward primer 5’-AAGCAGGGATGATGTTCTGG-3’; reverse primer 5’-ATGCCTCCTGTACCACCAAC-3’). All samples were stored at -80°C until downstream analyses was performed. For the microarray experiments individual biotin-labelled cRNA was synthesized by the GeneChip 3’ Express Kit (Affymetrix, Santa Clara, CA, USA). According to the manufacturer’s protocol the cRNA was fragmented and hybridized on Affymetrix GeneChip porcine 24 k Arrays. After a washing and staining procedure the arrays were scanned (Affymetrix, Santa Clara, CA, USA).

### Data analyses

In total, 15 of 16 arrays passed the appropriate quality control criteria as previously proposed [21]. The data was GC-RMA normalized (Log2). In order to improve statistical power by excluding inappropriate probe-sets [22], the data was filtered by the MAS5 algorithm. Those probe-sets expressed in less than 50% per experimental group were skipped. Further, those probe-sets which revealed a small SD (SD <0.20) were excluded from further data processing, because the corresponding transcripts were not likely to show altered mRNA abundances. Finally, probe-sets which showed a mean less than 2.5 were skipped. In order to evaluate relative changes of mRNA abundances, a variance analyses was performed (SAS Institute, Cary, NC, USA), including individual and combined effects represented by stress level, sex, slaughter batch, mixing group, and stress level*sex (V_ijkl_ = μ + stress level_i_ + sex_j_ + slaughter batch_k_ + mixing group_l_ + (stress level*sex)_ij_ + error_ijkl_). In order to account for multiple testing p-values were converted to a set of q-values [23]. The level of significance was set at p ≤0.05 and q ≤0.25. The raw data has been deposited in a MIAME compliant database, the National Center for Biotechnology Information Gene Expression Omnibus ( http://www.ncbi.nlm.nih.gov/geo) (accession number: [GSE44992]).

### Pathway analyses

The probe-sets were annotated by EnsEMBL Susscrofa 9 [24]. In order to unravel putative pathways associated with altered mRNA abundances in porcine PBMC cells, gene lists obtained from the microarray analyses were evaluated with ‘Ingenuity Pathway Analysis’ (IPA release winter 2012, Ingenuity Systems, Redwood City, CA, USA). The significance of association between dataset and pathway analysis was calculated according to the Benjamini-Hochberg correction implemented in IPA (p ≤0.05).

### Quantitative real-time PCR

Total transcript levels of selected target (*CCR1*, *CD14*, *TLR2*, *TLR4*, and *TREM1*) and reference genes (*HPRT1*, *PPIA*, *RPL32*) were quantified by real-time qPCR (Table 2). In total, 15 individual PBMC mRNA samples were analysed in duplicate. First-strand cDNA was synthesized from 2 μg of total RNA (n =15) using random primers and oligo d(T) 13VN in the presence of Superscript II reverse transcriptase (Invitrogen, Karlsruhe, Germany). The analyses were performed on a LightCycler 480 system using LightCycler 480 SYBR Green I Master (Roche, Mannheim, Germany). The amplification was conducted in duplicate according to manufacturer's instructions. Reactions were performed in a final volume of 10 μl using 5.0 μl of LightCycler 480 SYBR Green I Master, 2.0 μl of Aqua dest., 10 μM (0.5 μl) of each primer (Table 2) and 40 ng (2 μl) cDNA. The temperature profiles comprised an initial denaturation step at 95°C for 10 min and 40 cycles consisting of denaturation at 95°C for 15 s, annealing at 60°C for 10 s and extension/fluorescence acquisition at 72°C for 15 s. For all the assays threshold cycles were converted to copy numbers using a standard curve generated by amplifying serial dilutions of an external PCR standard (10^7^ - 10^2^ copies). At the completion of the amplification protocol, all samples were subjected to melting curve analyses and gel electrophoresis to verify the absence of any non-specific product. To account for variation in RNA input and efficiency of reverse transcription the calculated mRNA copy numbers were factorial normalized. In particular, a ratio between mean expression values of individual sample and its stress-group was computed for each of the three housekeeping genes. Subsequently, these ratios were averaged and used as normalization factor. The Fold change (FC) was computed as a ratio between normalized expression values of HS and LS samples. Data were analysed using the PROC MIXED, including effects of stress level, sex, slaughter batch, mixing group, and stress level*sex (SAS Institute, Cary, NC). The level of significance was set at p ≤0.05.

**Table 2:** **Primer used to verify microarray experiments by qPCR**

Gene symbol	Sequence 5' - 3' For	Sequence 5' - 3' Rev	Size (bp)
CCR1	CATTCCAGAAGATTGGGACAA	TGGCTCCAGGCTCATAGTAGA	182
CD14	GAGGTGGCAGAGTTCAAAGAG	CATGGTCGATAAGGTCCTCAA	196
TLR2	TAAGTTGAAGACGCTCCCAGA	ACAGGAAGTCACAGGAGCAGA	167
TLR4	CTCTGCCTTCACTACAGAGA	TTGAGTCGTCTCCAGAAGAT	323
TREM1	GGGAGAGACCCTGAATGTGA	ATCTTCCCCACCTGGACTTTA	156
HPRT1*	GTGATAGATCCATTCCTATGACT	TGAGAGATCATCTCCACCAATTA	104
PPIA*	GATTTATGTGCCAGGGTGGT	CTTGGCAGTGCAAATGAAAA	179
RPL32*	AGCCCAAGATCGTCAAAAAG	TGTTGCTCCCATAACCAATG	165

*CCR1* – chemokine (C-C motif) receptor 1; *CD14* – CD14 molecule; *TLR2* – toll-like receptor 2; *TLR4* – toll-like receptor 4; *TREM1* – triggering receptor expressed on myeloid cells 1 precursor; *HPRT1* – hypoxanthine phosphoribosyltransferase 1; *PPIA* – peptidylprolylisomerase A (cyclophilin A); *RPL32* – ribosomal protein 32; * reference gene.

## Results

The samples used in this study were selected from a larger animal experiment [5]. In order to study transcriptional responses and to obtain molecular markers for stress levels in pig herds, different levels of psychosocial stress were induced by mixing unfamiliar pigs with different temperaments. Animals were assigned as either psychosocially high stressed (HS; n =8) or psychosocially low stressed (LS; n =8) to create two microarray experimental groups to analyse their transcriptional patterns in porcine PBMC. Mixing unfamiliar pigs affected physical and physiological parameters of psychosocial stress in the expected way: The skin lesion score was higher in HS than in LS animals and the physiological stress parameters creatine kinase activity and plasma cortisol level were elevated in HS compared to LS animals (Table 1). These issues reflect damage to muscle fibres due to strenuous physical activity and the resulting adrenal response due to psychosocial stress, respectively. Interestingly, differences between the measured parameters were sex-independent.

In total the microarray analyses identified 12,830 expressed probe-sets (~53%) according to MAS5 filtering. Further filtering steps based on the variability and strength of expression revealed 8,029 probe-sets for further analyses. These probe-sets represented 5,776 genes [24].

### Unaltered transcriptional patterns due to stress level

The analysis revealed 706 probe-sets with an altered mRNA abundance at p ≤0.05 that correspond to q-values ranging between 0.24 and 0.36. Indeed, only 2 probe-sets (Ssc.13877.1.A1_at and Ssc.3445.1.S1_at, annotated as *Trip11* and *ARL4C*, respectively) appeared to be altered in HS samples compared to LS samples at q <0.25. However, in order to get a first hint about transcriptional differences due to stress response, we uploaded this particular gene list (p ≤0.05; q ≤0.36) to IPA. HS and LS samples showed deviations within numerous immune pathways (HS > LS), including TREM1 signalling, dendritic cell maturation, Toll-like receptor signalling, and IL-6 signalling. Thus, these processes might represent transcriptional alterations which were only pronounced in a particular subgroup of the dataset. Thereupon, we examined the variance component stress level*sex to unravel the subtle modifications of the gene expression machinery.

### Sex-dependent mRNA alterations due to stress level

**Figure 2:**
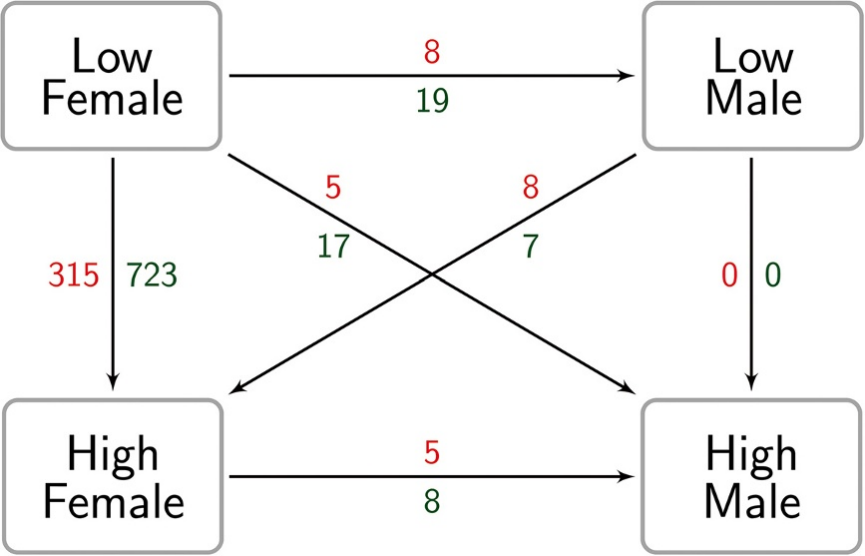
**Numbers of probe-sets showing altered mRNA abundances according to stress level and sex.** Probe-sets differ between females, but not between male animals. Red and green numbers indicate higher and lower transcript abundances, respectively (e.g. 315 probe-sets showed an increased mRNA abundance in HS-females compared to LS-females).

The variance analyses revealed differences in mRNA abundances due to stress level interacting with sex (Figure 2). In particular, relevant stress-dependent alterations were only found between female samples: Comparing HS-females and LS-females 1,038 probe-sets differed significantly (315 probe-sets: HS-females > LS-females). The magnitude of differential expression (fold change - FC) ranged between 18.8 (HS-females > LS-females) and -4.2 (HS-females < LS-females). According to IPA a set of immunological pathways were found to differ due to stress level among female samples. The top pronounced pathways which differed in transcript levels are displayed in Table 3 and Additional file 1. Genes associated with TREM 1 signalling, dendritic cell maturation, IL-6 signalling, Toll-like receptor signalling, and IL-8 signalling showed an increased mRNA abundance in HS-females. No single pathway was found to be increased in LS-females using Benjamini-Hochberg multiple testing corrections.

**Table 3:** **Ingenuity pathway analysis of transcripts with higher expression between High-stressed females and Low-stressed females**

Canonical pathway	Expression	p-value	Involved genes with altered mRNA abundances												
Dendritic cell maturation	HSF > LSF	6.20E-04	Gene symbol	CD1D	ICAM1	IL1B	IL1RN	JAK2	MYD88	TLR2	TLR4	TNFRSF1A	TNFRSF1B	TRD@	TYROBP
			Fold Change	+1.74	+1.56	+2.35	+3.07	+1.69	+1.85	+1.82	+3.71	+1.43	+1.79	+1.81	+1.92
IL-6 signaling	HSF > LSF	7.69E-03	Gene symbol	A2M	CD14	CSNK2A1	IL1B	IL1RN	JAK2	TNFRSF1A	TNFRSF1B				
			Fold Change	+3.00	+3.89	+1.38	+2.35	+3.07	+1.69	+1.43	+1.79				
IL-8 signaling	HSF > LSF	4.67E-02	Gene symbol	GNG10	ICAM1	MYL2	PTGS2	RHOQ	SRC	TEK	VASP				
			Fold Change	+1.70	+1.56	+1.49	+2.62	+2.13	+1.51	+2.12	+1.26				
Toll-like receptor signaling	HSF > LSF	1.83E-02	Gene symbol	CD14	LY96	MYD88	TLR2	TLR4							
			Fold Change	+3.89	+1.43	+1.85	+1.82	+3.71							
TREM 1 signaling	HSF > LSF	3.22E-05	Gene symbol	CASP1	ICAM1	IL1B	JAK2	MYD88	TLR2	TLR4	TREM1	TYROBP			
			Fold Change	+1.70	+1.56	+2.35	+1.69	+1.85	+1.82	+3.71	+2.80	+1.92			

P-value: significance of association between dataset and IPA according to Benjamini-Hochberg multiple testing correction; Details regarding involved genes were displayed in Additional file 1.

Despite pronounced differences in their stress level (Table 1), the comparisons between male samples revealed no alterations regarding their expression profiles (HS-males = LS-males). Moreover, the remaining comparisons between females and males revealed only marginal transcriptional differences (Figure 2).

**Figure 3:**
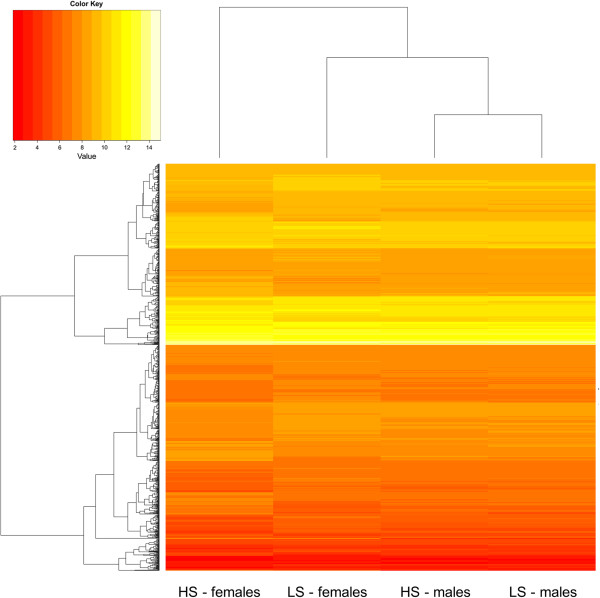
Heat-map considering the altered probe-sets (rows) due to the variance component stress level*sex (columns). The clustering revealed HS-females as most different from LS-females and male samples.

All probe-sets found to differ due to the variance component stress level*sex were clustered (Figure 3) to estimate the relationship of the appearing subgroups (HS-females, HS-males, LS-females, and LS-males). The analyses identified the transcriptional responses of HS-female samples as most distant compared to LS-females and male samples. Hence, the alteration of immunological pathways between female samples was considered a specific transcriptional response of HS-females.

### Alterations in mRNA abundances of selected cell surface receptors

A variety of transcripts encoding immune receptors (*CCR1, CCRL2, CD1D, CD14, CD19, CR2, CXCR7, IL13RA1, IL27RA, IL6R, IL7R, TLR2, TLR4, TNFRSF1, TNFRSF1B, TRAF5, TREM1*) showed different mRNA abundances dependent on the affiliation to either the HS or LS group in females but not in males (Table 4). In order to validate the microarray experiment, 5 selected transcripts encoding cell surface receptors associated with immune functions were analysed by qPCR: *CCR1*, *CD14*, *TLR2*, *TLR4*, and *TREM1* (Table 5). Regarding the microarray analysis, the mean expression values (log2) of the selected target and reference genes ranged between 3.94 and 15.06. Thus, the subset represented the distribution of expression values and highlighted the independence of expression variability and logarithmised mRNA abundances. Within the microarray experiment all selected transcripts showed significantly altered mRNA abundances between females (HS-females > LS-females), but not between male samples (q-values >0.25). The qPCR data confirmed the sex-specific expression pattern: 4 of 5 selected transcripts encoding cell surface receptors showed increased mRNA abundances in HS-females compared to LS-females. No alterations between the male samples were detected. Also the fold changes revealed a reliable dimension. Between microarray and qPCR data the correlation coefficients were highly significant and ranged between 0.87 and 0.93. Taken together, the qPCR analyses indicated a reproducible analysis.

**Table 4:** **Selected transcripts encoding immune receptors**

Gene symbol	Comparison	Microarray			
		p-value	q-value	Expression	FC
CCRL2	Females	<0.05	<0.25	HSF > LSF	+2.22
	Males	>0.10	>0.25	HSM = LSM	+1.02
CD1D	Females	<0.01	<0.25	HSF > LSF	+1.74
	Males	>0.10	>0.25	HSM = LSM	+1.19
CD19	Females	<0.05	<0.25	HSF < LSF	-2.70
	Males	>0.10	>0.25	HSM = LSM	-1.05
CR2	Females	<0.01	<0.25	HSF < LSF	-3.13
	Males	>0.10	>0.25	HSM = LSM	+1.01
CXCR7	Females	<0.05	<0.25	HSF < LSF	-2.83
	Males	>0.10	>0.25	HSM = LSM	-1.54
IL13RA1	Females	<0.01	<0.25	HSF > LSF	+2.55
	Males	>0.10	>0.25	HSM = LSM	+1.04
IL27RA	Females	<0.01	<0.25	HSF < LSF	-1.38
	Males	>0.10	>0.25	HSM = LSM	+1.04
IL6R	Females	<0.05	<0.25	HSF < LSF	-1.34
	Males	>0.10	>0.25	HSM = LSM	+1.08
IL7R	Females	<0.05	<0.25	HSF < LSF	-1.84
	Males	>0.10	>0.25	HSM = LSM	+1.01
TNFRSF1A	Females	<0.05	<0.25	HSF > LSF	+1.43
	Males	>0.10	>0.25	HSM = LSM	-1.03
TNFRSF1B	Females	<0.05	<0.25	HSF > LSF	+1.79
	Males	>0.10	>0.25	HSM = LSM	-1.12
TRAF5	Females	<0.05	<0.25	HSF < LSF	-1.83
	Males	>0.10	>0.25	HSM = LSM	+1.12

Significance level was set at p <0.05 and q <0.25; *CCRL2* – chemokine (C-C motif) receptor-like 2; *CD1d* – CD1d molecule; *CD19* – CD19 molecule; *CR2* – complement component receptor 2; *CXCR7* – chemokine (C-X-C motif) receptor 7; *IL13RA1* – interleukin 13 receptor, alpha 1; *IL27RA* – interleukin 27 receptor, alpha; *IL6R* – interleukin 6 receptor; *IL7R* – interleukin 7 receptor; *TNFRSF1A* – tumor necrosis factor receptor superfamily, member 1A; *TNFRSF1B* – tumor necrosis factor receptor superfamily, member 1B; *TRAF5* – TNF receptor-associated factor 5; FC – Fold change.

**Table 5:** **Comparison of microarray and quantitative PCR (qPCR) results for selected transcripts, encoding immune receptors**

Gene symbol	Comparison	Microarray				Real-time PCR #			Correlation ##
		p-value	q-value	expression	FC	p-value	expression	FC	coefficient
CCR1	Females	<0.05	<0.25	HSF > LSF	+2.60	<0.10	HSF = LSF	+1.81	0.93***
	Males	<0.05	>0.25	HSM = LSM	+1.75	>0.10	HSM = LSM	+1.23	
CD14	Females	<0.05	<0.25	HSF > LSF	+3.89	<0.05	HSF > LSF	+1.88	0.87***
	Males	<0.05	>0.25	HSM = LSM	+2.53	>0.10	HSM = LSM	+1.11	
TLR2	Females	<0.01	<0.25	HSF > LSF	+1.82	<0.05	HSF > LSF	+1.34	0.87***
	Males	<0.05	>0.25	HSM = LSM	+1.44	>0.10	HSM = LSM	+1.10	
TLR4	Females	<0.05	<0.25	HSF > LSF	+3.71	<0.05	HSF > LSF	+1.82	0.88***
	Males	<0.10	>0.25	HSM = LSM	+1.85	>0.10	HSM = LSM	+1.15	
TREM1	Females	<0.01	<0.25	HSF > LSF	+2.80	<0.01	HSF > LSF	+3.51	0.90***
	Males	<0.01	>0.25	HSM = LSM	+2.18	<0.10	HSM = LSM	+2.09	

Significance level was set at p <0.05 and q <0.25; FC - Fold change; # Values were calculated by factorial normalization on *RPL32*, *PPIA* and *HPRT1* expression values; ## correlation of normalized expression values was calculated by Spearman (*** p < 0.0001).

## Discussion

Due to mixing unfamiliar pigs, different levels of psychosocial stress were induced. Transcriptional shifts in PBMC were evaluated via a microarray experiment to gain insights into the molecular routes directing effects of psychosocial stress to the immune status.

### HS-females showed transcriptional responses to psychosocial stress

Studies in various mammalian species have clearly demonstrated that the immune response is sexually dimorphic [25]. Here, the complex interactions between sex steroid environment, HPA axis hormones and the immune system appeared to be involved as reviewed elsewhere [26]. In particular, adult female mammals have greater humoral and cellular immunity than males. In male castrates the lack of androgen release interrupts the negative feedback within the HPA axis and might culminate in immunosuppressive effects following increased endogenous cortisol. Moreover, castration causes various immune alterations, including alterations in organ weight of both thymus and spleen [27, 28] and the number of mature T-cells [28]. Hence, in our study remarkable alterations of porcine immune transcripts appeared due to the interaction between stress level and sex. Interestingly, the cluster analyses (Figure 3) identified the HS-females as most separated among the female and male samples. Thus, our findings suggest that immune-associated adaptations occurred mainly in HS-females, where the resulting differences in gene expression are likely to represent transient rather than persistent processes. One may argue that the observed mRNA alterations might have their reason in the degree of sexual maturity. In fact, levels of sexual steroid hormones involved in the oestrous cycle impact on immune cell distribution [29–31]. However, both 17β-estradiol (HS-females: 30.23 ± 7.90 pg/ml; LS-females: 26.52 ± 9.41 pg/ml) and progesterone (HS-females: 0.40 ± 0.20 ng/ml; LS-females: 0.29 ± 0.21 ng/ml) were found to be unaltered.

### Stress and immunity

In order to understand apparently contrary results in the field of psychoneuroimmunology, a multidimensional approach was proposed, involving at least the factors social status, age, genetic background, investigated tissue, time of day, and particularly the stressor characteristics [32]. Dhabhar [32] concluded that a stressor mediated by endogenous hormones in physiological concentrations (e.g. cortisol [33]) acting on an immune compartment naturally enriched with immune cells (e.g. blood, PBMC) will reveal immune enhancing effects. Indeed, the significantly increased molecular routes revealed by the pathway analyses (HS-females > LS-females) indicate that high psychosocial stress involves a preparation of the immune system in terms of an immune enhancement. However, it appears to be difficult to evaluate whether the observed mRNA alterations reflect bio-positive side effects in terms of immune resistance and immune tolerance [34, 35].

It has been shown in numerous studies, that a response to psychosocial stress cumulated in adaptive mechanisms of different tissues and organs [36], thereby affecting the immune system to face environmental challenges [7, 37, 38]. Consequently, the immune pathways found to be altered in HS-females compared to LS-females (Table 3) corresponded to both the innate and the adaptive immune mechanisms, including processes associated to pattern recognition and inflammation. In particular, cell surface receptors (Table 4) showed both increased and decreased mRNA abundances in HS-females, indicating a fine tuning of the immune system as response to the psychosocial challenge. Altering specific cell surface receptors in their transcript yields suggest that the socially stressed organism adapts its signal transduction in order to parry possible infections due to injuries while fighting [39].

Due to the intensive linkage between brain, behaviour, endocrine system, and immune system [40], aggression has been found to activate both the hypothalamic-pituitary-axis (HPA) and the sympatho-adrenomedullar (SAM) system [5, 41]. Subsequently, adaptive mechanisms are transmitted via humoral and cellular mediators (i.e. chemokines and cytokines) whose transcription and release are generated in narrow temporal confines in order to modulate various immune actions. Consequently, in our study the psychosocially high-stressed pigs had increased plasma cortisol at slaughter, whereas transcriptional alterations of genes encoding secreted immune factors were observed only marginally (e.g. IL1B, IL1RN). These findings are consistent with previous results in rodents, when an exposure to a dominant mouse led to elevated corticosterone levels but unaffected circulating cytokines at sampling time [42].

These observations lead to the general remark, that mRNA alterations of secreted immune factors contribute to the phenomenon ‘stress response’ while possible differences in mRNA abundances of cell surface receptors partly reflect the degree of ‘stress responsiveness’. Thus, here we observed differences in both ‘stress response’ and ‘stress responsiveness’.

## Conclusions

In order to gain knowledge of molecular routes linking stress reaction and immune status, the transcriptional response of PBMC to psychosocial stress was evaluated. The stress reaction impacted on transcripts of immunocompetent cells and appeared to be gender specifically in females only. Similar levels of steroid hormones among the high and low stressed groups might indicate that not direct effects of sexual hormones on the endocrine and immune systems are relevant for the gender specific effects, but probably effects on the cognition and perception of psychosocial stressors.

The expression profiles of psychosocially high-stressed female animals were associated with transcriptional shifts of pathways of acquired and innate immunity. Moreover, besides transcripts of immune effectors the analyses highlighted mRNAs of various cell surface receptors as stress-sensitive. The stress-dependent expression patterns indicate an alerting of the immune system in terms of both response and responsiveness mediated by an increased delivery of effector molecules and an installation of receptors.

## Electronic supplementary material

Additional file 1: **Ingenuity pathways and metadata of involved genes (p values, q values, Fold changes) differing between HS-females and LS females.** (XLS 32 KB)

## Abbreviations

PBMC: Peripheral blood mononuclear cells
HSF: High stressed females
LSF: Low stressed females
HSM: High stressed males
LSM: Low stressed males.
